# *Aspergillus fumigatus* melanins: interference with the host endocytosis pathway and impact on virulence

**DOI:** 10.3389/fmicb.2012.00440

**Published:** 2013-01-18

**Authors:** Thorsten Heinekamp, Andreas Thywißen, Juliane Macheleidt, Sophia Keller, Vito Valiante, Axel A. Brakhage

**Affiliations:** ^1^Department of Molecular and Applied Microbiology, Leibniz Institute for Natural Product Research and Infection Biology – Hans Knöll InstituteJena, Germany; ^2^Department of Microbiology and Molecular Biology, Friedrich Schiller UniversityJena, Germany

**Keywords:** *Aspergillus fumigatus*, melanin, virulence, apoptosis, phagocytes, endocytosis

## Abstract

The opportunistic human pathogenic fungus *Aspergillus fumigatus* produces at least two types of melanin, namely pyomelanin and dihydroxynaphthalene (DHN) melanin. Pyomelanin is produced during tyrosine catabolism via accumulation of homogentisic acid. Although pyomelanin protects the fungus against reactive oxygen species (ROS) and acts as a defense compound in response to cell wall stress, mutants deficient for pyomelanin biosynthesis do not differ in virulence when tested in a murine infection model for invasive pulmonary aspergillosis. DHN melanin is responsible for the characteristic gray-greenish color of *A. fumigatus* conidia. Mutants lacking a functional polyketide synthase PksP, the enzyme responsible for the initial step in DHN-melanin formation, i.e., the synthesis of naphthopyrone, produce white spores and are attenuated in virulence. The activity of PksP was found to be essential not only for inhibition of apoptosis of phagocytes by interfering with the host PI3K/Akt signaling cascade but also for effective inhibition of acidification of conidia-containing phagolysosomes. These features allow *A. fumigatus* to survive in phagocytes and thereby to escape from human immune effector cells and to become a successful pathogen.

## Melanins in fungi

Among airborne acquired fungal infections of immunocompromised humans, diseases caused by *Aspergillus fumigatus* clearly predominate (Brakhage, 2005). Today, it is generally accepted that multiple factors contribute to virulence of *A. fumigatus*, e.g., its thermotolerance, its versatile metabolism, or the production of toxins and other secondary metabolites. Such a secondary metabolite is dihydroxynaphthalene (DHN) melanin, accounting for the gray-green pigmentation of conidia.

The term melanin originates from the greek word *mèlas* meaning black. Melanins are broadly defined as brown to black pigments of high molecular mass derived from oxidative polymerization of phenolic precursors (Riley, 1997). The ability to produce melanin is a common feature of many organisms and these pigments are attributed with a wide variety of beneficial functions: They protect organisms against different exogenous stresses, e.g., UV-irradiation, elevated temperatures, reactive oxygen species (ROS), and also against microbial lytic enzymes and defensins (Doering et al., 1999; Langfelder et al., 2003; Nosanchuk and Casadevall, 2006). Melanins can bind metal ions and free electrons and thus function as physiological redox buffer (Jacobson, 2000). In fungi, melanins are often associated with the cell wall and are normally extracellularly localized. Even the synthesis often does not occur in the cytoplasm due to the fact that intermediates are potentially toxic to the producing organism. Cell wall bound melanin can be visualized by transmission electron microscopy as an electron-dense outer layer or associated with a matrix outside of the cell wall (Pihet et al., 2009). Melanins also contribute to the structure of spores. *A. fumigatus* wild-type conidia show ornamentation on their surface whereas melanin-free conidia possess a characteristically smooth surface (Jahn et al., 1997). Remarkably, by contrast to pigmentless white conidia, other conidial color mutants of *A. fumigatus*, that produce yellow, reddish, or brown pigments, show wild-type like ornamentation (Sugareva et al., 2006; Schmaler-Ripcke, pers. communication). This review focus on the two pigments currently known to be produced by *A. fumigatus*: DHN melanin and pyomelanin. Recent findings on their biosynthesis and their role in virulence will be discussed.

## Pyomelanin in *A. fumigatus*

**Figure 1:**
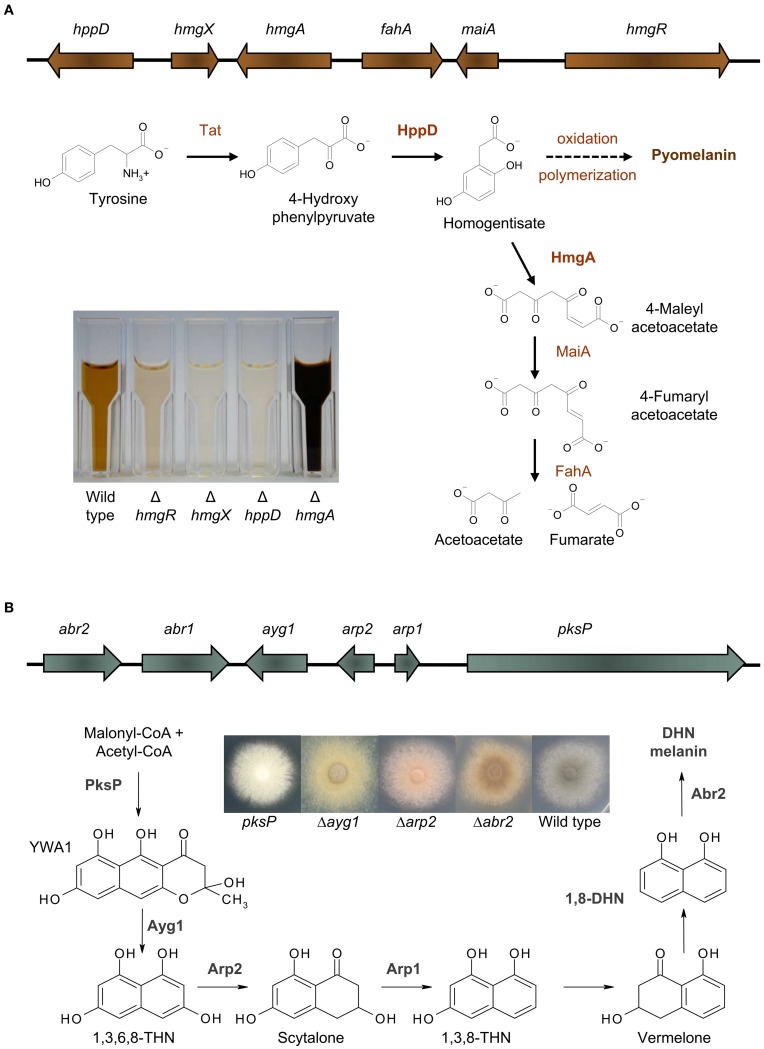
**Melanins in *A. fumigatus.* (A)** Pyomelanin: the tyrosine degradation pathway and genomic organization of the genes involved in tyrosine degradation and pyomelanin formation. The degradation of tyrosine starts with the formation of *p*-hydroxyphenylpyruvate (*p*HPP), which is then converted to HGA by the *p*-hydroxyphenylpyruvate dioxygenase (HppD). HGA is either degraded enzymatically by maleylacetoacetate isomerase (MaiA) and fumarylacetoacetate hydrolase (FahA) to fumarate and acetoacetate, compounds of the primary metabolism, or homogentisate polymerizes oxidatively to pyomelanin. Pyomelanin production is shown in supernatants of different *A. fumigatus* strains cultivated in minimal medium with addition of tyrosine. **(B)** DHN melanin: the DHN-melanin biosynthesis pathway and genomic organization of the genes involved in DHN-melanin biosynthesis [adapted from Langfelder et al. (2003) and Tsai et al. (2001)]. Starting from acetyl-CoA and malonyl-CoA, PksP produces the heptaketide naphthopyrone YWA1, which is shortened by hydrolytic activity of Ayg1 to 1,3,6,8-tetrahydroxy naphthalene (THN). This pentaketide undergoes reduction, mediated by the THN reductase Arp2 and dehydration by the scytalone dehydratase Arp1. Finally, the laccase Abr2 catalyses oxidative polymerization of 1,8-DHN to form the final pigment (Tsai et al., 2001; Fujii et al., 2004; Sugareva et al., 2006). The picture shows the color of conidia of different DHN-melanin biosynthesis mutants.

Recently it was shown, that *A. fumigatus* excretes a brown compound, when L-tyrosine or L-phenylalanine is present in the medium (Schmaler-Ripcke et al., 2009). This water soluble, dark brown pigment is synthesized extracellularly and is able to bind to the surface of hyphae. Pyomelanin production in *A. fumigatus* occurs by oxidative polymerization of homogentisate (HGA), an intermediate of the tyrosine degradation pathway (Figure 1A). Several essential genes for tyrosine degradation are arranged in a cluster in the genome of *A. fumigatus*. The cluster consists of the genes *hppD, hmgX, hmgA, fahA, maiA*, and *hmgR*, which are coordinately transcribed. The generation of loss of function mutants as well as GFP-tagged strains allowed a detailed functional characterization of specific genes of this cluster and their contribution to pyomelanin biosynthesis (Schmaler-Ripcke et al., 2009; Keller et al., 2011). HppD mediates the conversion of *p-hydroxyphenylpyruvate* (*p*HPP) to HGA and therefore deletion of *hppD* resulted in the disability of HGA synthesis and consequently impaired pyomelanin formation. By contrast, *hmgA* gene deletion prevents cleavage of the aromatic ring of HGA and its conversion to 4-maleylacetoacetate, resulting in HGA accumulation and increased pyomelanin formation. Another crucial role in tyrosine degradation and pyomelanin biosynthesis is played by HmgX. Deletion of *hmgX* resulted in the disability of pyomelanin production, comparable to that of the Δ*hppD* mutant. Although the precise function of HmgX is still unclear, it was shown that the presence of this cytoplasmic protein is important for the enzymatic conversion of *p*HPP to HGA. HmgX might function as an accessory factor to mediate specific HppD activity. The clustered organization and the uniform transcriptional activation in the presence of tyrosine, suggested the existence of a common regulator of the genes involved in tyrosine degradation. HmgR contains a Zn(II)2Cys6-DNA binding domain. The nuclear localization of HmgR as well as its necessity for tyrosine-induced transcription of the cluster genes, identified HmgR as the transcriptional activator of the tyrosine degradation cluster (Keller et al., 2011).

## Connection of pyomelanin to cell wall integrity and role in virulence

It was intriguing to speculate whether pyomelanin contributes to virulence. Pyomelanin exerts a protective role against ROS (Schmaler-Ripcke et al., 2009). Germlings of the non-pyomelanin producing mutant Δ*hppD* showed increased susceptibility against hydrogen peroxide and the thiol-oxidizing agent diamide in presence of tyrosine. The observation of a protective role for pyomelanin is consistent with the finding that cell wall stress signaling via the MpkA cell wall integrity pathway in *A. fumigatus* influences pyomelanin formation (Valiante et al., 2009). Transcription of the tyrosine degradation gene cluster was increased under cell wall stress (Jain et al., 2011). Furthermore a post-transcriptional regulation was discussed, since deletion mutants of the MpkA pathway showed slightly reduced HmgA activity and consistently increased pigment formation when cultivated in the presence of tyrosine (Valiante et al., 2009). It is hypothesized that this MAP kinase module regulates the balanced activity of enzymes involved in tyrosine degradation and enables the fungus to accumulate pyomelanin as a defense compound upon cell wall stress.

Interestingly, several genes of the tyrosine degradation cluster were found to be differentially regulated when *A. fumigatus* was confronted with immune cells. In response to immature dendritic cells, four genes of the cluster (*hppD, hmgX, hmgA, fahA*) were significantly upregulated (Morton et al., 2011). These genes also showed increased transcription when *A. fumigatus* was confronted with human neutrophils (McDonagh et al., 2008). Hence, pyomelanin formation might be involved in escape from the immune system and survival of the fungus. Consistently, in a microarray analysis all six genes of the tyrosine degradation cluster were found to be upregulated *in vivo* in infected mouse lung tissue (McDonagh et al., 2008) and transcripts of *hppD* and *hmgA* were confirmed during infection by RT-PCR (Keller et al., 2011). Since *A. fumigatus* can secrete several proteases (Behnsen et al., 2010; Hartmann et al., 2011; Wartenberg et al., 2011) it is possible that tyrosine is available during infection and activates transcription of the tyrosine degradation gene cluster (Keller et al., 2011). However, glucose or nitrogen starvation conditions were also found to be sufficient for induction of expression of genes involved in tyrosine degradation (Keller et al., 2011), although these starvation conditions did not result in the accumulation of pyomelanin. Therefore, the finding that genes of the tyrosine degradation cluster are transcribed *in vivo* during infection does not necessarily indicate an essential role for pyomelanin in fungal pathogenicity. It is unclear whether *A. fumigatus* is able to produce pyomelanin in sufficient amounts at the site of infection and whether this compound accumulates around the hyphae in a way to protect against attacks of the immune system. The mutants Δ*hppD* and Δ*hmgR*, that are not able to produce pyomelanin, caused no altered mortality in an infection model for pulmonary aspergillosis using corticosteroid treated mice (Keller et al., 2011). This implies that hyphal pyomelanin is dispensable for *A. fumigatus* virulence, at least in the infection model employed.

## DHN melanin in *A. fumigatus*

The most prevalent melanin produced by fungi is DHN melanin (Wheeler and Bell, 1988). In *A. fumigatus*, DHN melanin accounts for the typical gray-greenish color of its conidia. By applying classical mutagenesis, *A. fumigatus* conidial color mutants producing brown, yellow, reddish, or white conidia were generated. The first gene identified to be essential for conidial pigment formation was *pksP*, encoding a polyketide synthase and representing the core gene of the identified melanin biosynthesis cluster (Jahn et al., 1997; Langfelder et al., 1998; Tsai et al., 1998). Besides *pksP*, this cluster contains the genes *ayg1, arp1, arp2*, and *abr2* that are all necessary to produce DHN melanin in *A. fumigatus* (Figure 1B). A similarity search using BLASTP (Altschul et al., 1990) revealed that a homolog for PksP is found in most aspergilli sequenced up to date with the exception of *Aspergillus terreus*. The same situation is found for Ayg1 mediating the next step for DHN-melanin biosynthesis. The protein identity for PksP and Ayg1 homologs for *Neosartorya fischeri, Aspergillus clavatus, Aspergillus flavus, Aspergillus oryzae, Aspergillus niger*, and *Aspergillus nidulans* is at least 60% whereas for *A. terreus* only proteins with identities of 42% (PksP) or 23% (Ayg1) could be identified. For all other proteins involved in DHN-melanin biosynthesis in *A. fumigatus* unambiguous homologs are only found in close relatives of *A. fumigatus*, i.e., *N. fischeri* and *A. clavatus*. In these species, the genes are also organized in a cluster similar to *A. fumigatus*. In the genomes of other aspergilli, such a cluster can not be found. Therefore, it is still a matter of debate whether other aspergilli, harboring a homolog for PksP and Ayg1, are also able to produce DHN melanin. In this regard, studies involving tricyclazole, an inhibitor of THN-reductases, imply that other aspergilli do not produce DHN melanin (Wheeler and Bell, 1988).

## Regulation of DHN-melanin biosynthesis

The regulation of DHN-melanin biosynthesis is only understood in part and the transcription factor regulating DHN-melanin biosynthesis has still not been identified yet. The promoter of the *pksP* gene harbors binding sites for a stress response element (STRE), and a cAMP response element. Additionally, putative binding sites for the transcription factors AbaA and StuA are present (Sugareva et al., 2006). Regulation of *pksP* transcription is connected to cAMP signaling via protein kinase A (PKA). This is supported by several findings: (1) mutants for the adenylate cyclase AcyA are severely affected in growth and only produce few spores which remain white; (2) deletion mutants for the G protein alpha subunit GpaB show delayed pigmentation of conidia (Liebmann et al., 2003, 2004); (3) in strains with elevated PKA activity, either by overproducing the catalytic subunit PkaC1 or deleting the gene *pkaR* encoding the regulatory subunit PkaR, *pksP* transcription was increased and these mutants consistently produced a dark mycelial pigment (Zhao et al., 2006; Grosse et al., 2008). By contrast, in Δ*gpaB* and Δ*pkaC1* mutants transcription of *pksP* was reduced (Liebmann et al., 2003). The global regulator LaeA contributes to the regulation of genes involved in production of secondary metabolites. As part of the *velvet complex*, LaeA is suggested to regulate transcription *via* chromatin remodeling (Palmer and Keller, 2010). However, whether LaeA is involved in transcriptional regulation of DHN-melanin biosynthesis genes is discussed controversially. Whereas conidial pigmentation in Δ*laeA* mutants is unaffected, transcription of *pksP* was found increased (Bok et al., 2005) or decreased (Sugui et al., 2007) under hyphal growth conditions. Both studies report that production of a mycelial pigment is inhibited in strain Δ*laeA*. Transcription of *pksP* occurs in all morphotypes, though the other genes of the DHN cluster are mainly transcribed during conidiogenesis. For example, during spore formation, transcription of *abr2* increased 17-fold whereas *pksP* transcription increased only 2.5-fold (Sugareva et al., 2006). Finally, monitoring expression of a *pksP*-promoter-GFP construct revealed transcription in hyphae isolated from lungs of infected mice (Langfelder et al., 2001).

## DHN melanin as a virulence determinant

Pigmentless conidia of *A. fumigatus* were more effectively killed by macrophages and the *pksP* mutant was attenuated in virulence in mice infection studies (Jahn et al., 1997, 2000, 2002; Langfelder et al., 1998; Tsai et al., 1998). By contrast, infection with Δ*arp2*, Δ*abr2*, and Δ*ayg1* conidia displayed the same virulence as wild-type conidia in mice (Tsai et al., 1999; Sugareva et al., 2006; Schmaler-Ripcke, pers. communication). Remarkably, the infection models employed to assess virulence of *A. fumigatus* melanin mutant strains differed considerably. Where in the older studies (Jahn et al., 1997; Langfelder et al., 1998; Tsai et al., 1998) conidia were injected *via* the lateral tail vein the more recent studies used an inhalation model to induce aspergillosis in mice. In the inhalation model, mice were immunosuppressed prior to infection using either cortisone acetate or cortisone acetate in combination with cyclophosphamide. In contrast to the leucopenic model, in mice treated solely with cortisone acetate recruitment of neutrophils and monocytes to the site of infection still occurs.

When sensitivity of *A. fumigatus* conidia against ROS was tested, *pksP* conidia were significantly more susceptible than wild-type conidia. However, this increased sensitivity was not found for the conidial color mutants Δ*ayg1*, Δ*arp2*, and Δ*abr2* (Sugareva et al., 2006; Schmaler-Ripcke, pers. communication). This finding suggests that polymerization products of DHN-melanin precursors, like YWA1, 1,3,6,8-THN, and 1,8-DHN are already able to scavenge ROS in a similar manner like DHN melanin. All these data imply that not DHN melanin itself but a shunt product, deriving from the first product naphthopyrone, synthesized by PksP, contributes to *A. fumigatus* virulence.

## Interference of DHN melanin with immune cells

Host immune cells produce ROS as defense mechanism, and both, DHN melanin and pyomelanin, protect the fungus against ROS. However, it is unlikely, that ROS produced by phagocytes play a significant role in killing *A. fumigatus*. The hypothesis that the *pksP* mutant is avirulent only due to its increased sensitivity toward host-derived ROS is rather questionable, since mutants for *Afyap1* and *skn7*, both acting as central fungal regulators mediating resistance against ROS, do not differ in virulence from wild-type conidia in a murine model of pulmonary aspergillosis (Lamarre et al., 2007; Lessing et al., 2007).

*A. fumigatus* produces several secondary metabolites that are known to interfere with host immune cells, for example fumagillin and helvolic acid that inhibit the function of neutrophils or the oxidative burst of macrophages (Mitchell et al., 1997; Fallon et al., 2010). The most prominent example, however, is gliotoxin that modulates the immune response and induction of apoptosis of host cells via activity of its unusual intramolecular disulfide bridge [reviewed in Scharf et al. (2012)]. Beside these toxins, an important role for DHN melanin and especially *pksP* was identified when the interaction of *A. fumigatus* conidia with immune effector cells was investigated. During infection, the host's immune system is confronted with several fungal morphotypes, namely resting conidia, swollen conidia, germlings, and hyphae. Recently, it was found that the conidial rodlet layer, consisting of the hydrophobin RodA renders fungal resting spores immunologically inert (Aimanianda et al., 2009). As soon as *A. fumigatus* conidia meet an appropriate environment, they start to swell leading to loss of the rodlet layer and the presentation of immunogenic surface structures. In resting conidia, melanin is part of the exterior surface of the cell wall and also contributes to the immunomodulatory capacity of *A. fumigatus* (Chai et al., 2010). By contrast to wild-type conidia and melanin ghosts that were only weak inducers of proinflammatory cytokine production, pigmentless conidia considerably trigger cytokine response, indicating that melanin is involved in masking fungal immunogenic structures. Following recognition by professional phagocytes that represent the first line of defense against *A. fumigatus*, the fungus is phagocytosed and the conidia-containing phagosomes then fuse with lysosomes. Usually, these phagolysosomes acidify and eliminate the ingested pathogen. However, *A. fumigatus* conidia are killed with different efficacies from phagocytes depending on their pigment. Wild-type conidia in the majority survived ingestion by macrophages whereas *pksP* mutant conidia were killed by 80% (Jahn et al., 2002). Recently, by dissecting the endocytotic pathway of phagocytes using confocal fluorescence microscopy it was shown that *A. fumigatus* is able to highly reduce acidification of phagolysosomes whereas the fusion of conidia-containing phagosomes with lysosomal vesicles was unaltered. This was true for several phagocytic cell types tested, namely murine-derived alveolar macrophages, human monocyte-derived macrophages, and human neutrophils (Thywißen et al., 2011). The same reduction was accomplished by phagocytosis of DHN-melanin ghosts and also by inhibition of the phagolysosomal vATPase activity *via* treatment with the specific vATPase inhibitor bafilomycin. Consistently, the *pksP* mutant completely failed to reduce phagolysosomal acidification. Conidia of Δ*ayg*1, Δ*abr2*, and Δ*arp2*, that produce DHN-melanin precursors, already reduced (but not to the extent of the wild-type) phagolysosomal acidification. This clearly indicates that the initial step in DHN-melanin biosynthesis catalysed by PksP is crucial and that the first DHN-melanin precursor, naphthopyrone, is already sufficient to reduce phagolysosomal acidification. Furthermore, other aspergilli that harbor a *pksP* homolog were consistently able to decrease phagolysosomal acidification. By contrast, *A. terreus*, which lacks a *pksP* homolog and does not produce naphthopyrone, was unable to inhibit phagolysosomal acidification (Thywißen et al., 2011; Slesiona et al., 2012).

**Figure 2:**
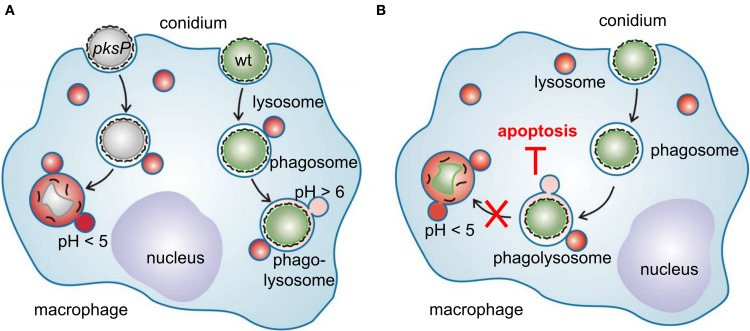
**Interference of *A. fumigatus* conidia with macrophages. (A)** Different intracellular fate of wild-type and *pksP* conidia. After recognition, swollen conidia are phagocytosed and fusion of the conidium-containing phagosome with lysosomal vesicles forms the phagolysosome. Wild-type conidia but not *pksP* conidia are able to inhibit phagolysosomal acidification (pH <5) and thereby prevent to be degraded by lytic proteins. **(B)** Intracellular presence of conidia is essential to protect macrophages from cell death. Conidia in acidified phagosomes do not exhibit anti-apoptotic properties.

The interaction of *A. fumigatus* with alveolar macrophages is summarized in a model (Figure 2) in which another feature of *A. fumigatus* to interfere with immune cells is depicted: *A. fumigatus* conidia inhibit apoptosis of macrophages by preventing activation of caspases of both the extrinsic and intrinsic apoptosis pathway (Volling et al., 2011). The anti-apoptotic ability by activation of the PI3K/Akt survival signaling pathway depends on the phagocytosis of conidia. Remarkably, activation of the PI3/Akt depends on DHN melanin, as only wild-type conidia and melanin ghosts but not *pksP* conidia induce phosphorylation of the PI3K/Akt kinase. Accordingly, induction of phagolysosomal acidification blocked the anti-apoptotic effect of wild-type conidia. Based on these findings, a model is proposed in which survival and escape from phagocytes occurs *via* a two step mechanism: (1) phagocytosed conidia inhibit phagolysosomal acidification to reduce fungal killing by macrophages and (2) conidia residing in non-acidified phagolysosomes inhibit phagocyte apoptosis to provide an intracellular niche for conidial survival.

## Conclusion

*A. fumigatus* produces at least two types of melanins: pyomelanin by polymerization of homogentisic acid, an intermediate of L-tyrosine catabolism, and DHN melanin *via* the polyketide synthase PksP. Transcription of genes essential for pyomelanin and DHN-melanin biosynthesis is detected during infection and both melanins protect the fungus against ROS. However, pyomelanin seems to be dispensable for fungal virulence at least in the murine infection models tested so far. By contrast, DHN melanin specifically interferes with functions of host phagocytes, allowing the fungus to generate an environment that allows its survival. In the near future, identification of the postulated transcription factor will be critical to further understand signaling pathways regulating *pksP* transcription during infection. Additionally, research will certainly focus on deciphering the exact mechanism by which *A. fumigatus* melanin inhibits assembly and activity of the vATP-ase of host phagocytes to inhibit acidification of phagolysosomes and thereby contributing to fungal pathogenicity. This might finally lead to the identification of new targets and antifungal compounds for therapeutic intervention.

### Conflict of interest statement

The authors declare that the research was conducted in the absence of any commercial or financial relationships that could be construed as a potential conflict of interest.
